# Down-regulation of *BCL2L13* renders poor prognosis in clear cell and papillary renal cell carcinoma

**DOI:** 10.1186/s12935-021-02039-y

**Published:** 2021-06-30

**Authors:** Fei Meng, Luojin Zhang, Mingjun Zhang, Kaiqin Ye, Wei Guo, Yu Liu, Wulin Yang, Zhimin Zhai, Hongzhi Wang, Jun Xiao, Haiming Dai

**Affiliations:** 1 Anhui Province Key Laboratory of Medical Physics and Technology, Institute of Health and Medical Technology, Hefei Institutes of Physical Science, Chinese Academy of Sciences, 350 Shushanhu Road, Hefei, 230031 Anhui China; 2 University of Science and Technology of China, Hefei, 230026 China; 3 Hefei Cancer Hospital, Chinese Academy of Sciences, Hefei, 230031 China; 4 Department of Hematology, The Second Affiliated Hospital of Anhui Medical University, Hefei, 230601 China; 5 Department of Oncology, The Second Affiliated Hospital of Anhui Medical University, Hefei, 230601 China; 6 Department of Urology, The First Affiliated Hospital of USTC, Division of Life Sciences and Medicine, University of Science and Technology of China, Hefei, 230001 China

**Keywords:** BCL-rambo, Cell death, Renal cancer, Prognosis, ANT

## Abstract

**Background:**

*BCL2L13* belongs to the *BCL2* super family, with its protein product exhibits capacity of apoptosis-mediating in diversified cell lines. Previous studies have shown that *BCL2L13* has functional consequence in several tumor types, including ALL and GBM, however, its function in kidney cancer remains as yet unclearly.

**Methods:**

Multiple web-based portals were employed to analyze the effect of *BCL2L13* in kidney cancer using the data from TCGA database. Functional enrichment analysis and hubs of *BCL2L13* co-expressed genes in clear cell renal cell carcinoma (ccRCC) and papillary renal cell carcinoma (pRCC) were carried out on Cytoscape. Evaluation of *BCL2L13* protein level was accomplished through immunohistochemistry on paraffin embedded renal cancer tissue sections. Western blotting and flow cytometry were implemented to further analyze the pro-apoptotic function of *BCL2L13* in ccRCC cell line 786-0.

**Results:**

*BCL2L13* expression is significantly decreased in ccRCC and pRCC patients, however, mutations and copy number alterations are rarely observed. The poor prognosis of ccRCC that derived from down-regulated *BCL2L13* is independent of patients’ gender or tumor grade. Furthermore, *BCL2L13* only weakly correlates with the genes that mutated in kidney cancer or the genes that associated with inherited kidney cancer predisposing syndrome, while actively correlates with *SLC25A4*. As a downstream effector of *BCL2L13* in its pro-apoptotic pathway, *SLC25A4* is found as one of the hub genes that involved in the physiological function of *BCL2L13* in kidney cancer tissues.

**Conclusions:**

Down-regulation of *BCL2L13* renders poor prognosis in ccRCC and pRCC. This disadvantageous factor is independent of any well-known kidney cancer related genes, so *BCL2L13* can be used as an effective indicator for prognostic evaluation of renal cell carcinoma.

**Supplementary Information:**

The online version contains supplementary material available at 10.1186/s12935-021-02039-y.

## Introduction

Kidney cancer remains one of the malignant tumors with the incidence increased notably in recent years [1]. It is composed of heterogeneous subtypes with histological and molecular abnormalities. Clear cell renal cell carcinoma (ccRCC) accounts for about 75% of all the cases, which is mainly characterized as constitutional chromosome 3p deletion, dysfunctional von Hippel-Lindau (*VHL*) gene and uncontrolled stabilization of hypoxia inducible factors (HIFs) by molecular features. Papillary renal cell carcinoma (pRCC), which occupies about 15% of the incidence, usually displays loss of chromosome Y, gain of chromosome 7 and/or chromosome 17. Moreover. different molecular characteristics are assigned to each pathological subtype, including both Type I and Type II pRCC [2, 3]. Chromophobe renal cell carcinoma (chRCC) and some other rare types make up the rest.

Mutations of *VHL* have been proved to be one of the typical genetic causes of kidney cancer, which often lead to stabilization of HIF1α and HIF2α, creating an illusion of pseudohypoxia in stricken renal tissues [4]. The accumulated HIF1α-HIF1β and HIF2α-HIF1β dimers will cause an elevation in growth factors, including platelet-derived growth factors (PDGFs) and vascular endothelial growth factors (VEGFs), which will prompt tumor angiogenesis [5, 6]. The VHL-HIF-VEGF axis represents one of the canonical pathways for renal carcinogenesis, while some other gene mutations or epigenetic changes have also been reported [7, 8], including mutations of polybromo-1 (*PBRM1*) [9], SET domain-containing 2 (*SETD2*) [10] and tuberous sclerosis complex 1/2 (*TSC1/2*) [11, 12]. Nevertheless, nearly 40% of the resected kidney cancer patients are confronted with risk of recurrence, the effective predictive markers are absent so far [13].

BCL-2-like protein 13 (BCL2L13), also termed BCL-rambo, is encoded by the *BCL2L13* gene [14]. It has manifested that BCL2L13 participates in drug resistance in several tumors. For example, BCL2L13 was found elevated in tumors like glioblastoma (GBM) and childhood acute lymphoblastic leukemia (ALL) [15, 16]. In GBM, BCL2L13 interacts with ceramide synthases 2 and 6 (CerS2/6), inhibiting the leakage of cytochrome c into cytoplasm [15]. Augmented BCL2L13, which takes part in L-asparaginase resistance, executes as an independent adverse prognostic factor in ALL [16]. On the other hand, BCL2L13 has been demonstrated to interact with adenine nucleotide translocator (ANT, encoded by *SLC25A4*), a component of mitochondrial permeability transition pore (MPTP), therefore promoting cytochrome c release from mitochondrial intermembrane space to cytoplasm, resulting in activation of the apoptotic caspase cascade [17].

The prognostic value of *BCL2L13* in kidney cancer is still unclearly. In this study, we profiled *BCL2L13* across 33 cancer types in the Cancer Genome Atlas (TCGA), and found that its mRNA expression is significantly reduced in ccRCC and pRCC. Moreover, low *BCL2L13* expression correlates with lessened survival probability of kidney cancer. It poses an attractive hypothesis that *BCL2L13* may be a promising prognosis marker for kidney cancer.

## Materials and methods

### University of California Santa Cruz (UCSC) genome browser and UCSC Xena

Protein–protein interaction (PPI) network analysis of *BCL2L13* was performed using UCSC genome browser (https://genome.ucsc.edu), which offers visualized interconnection of the concerned genes [18, 19]. *BCL2L13* mRNA and exon expression were completed on UCSC Xena platform (http://xena.ucsc.edu), complied with data from TCGA [20].

### Catalogue of Somatic Mutations in Cancer (COSMIC) and Open Targets Platform

The most frequent somatic mutations in ccRCC and pRCC tissues were queried by COSMIC cancer browser (http://cancer.sanger.ac.uk), showing the top 20 candidate genes curated from published research for each cancer type [21]. Heritable kidney cancer-predisposing syndrome related genes are searched from Open Targets Platform (https://www.targetvalidation.org). The overall target-disease association score is the harmonic sum aggregated from genetics, genomics, drugs, animal models and text mining data [22].

### University of Alabama Cancer Database (UALCAN) and cBioPortal for cancer genomics

The *BCL2L13* mRNA expression and corresponding effect on tumor prognosis were analyzed on UALCAN (http://ualcan.path.uab.edu/) [23–25]. Lymph node metastasis of renal cancer: N0, No regional lymph node metastasis; N1, Metastases in 1 to 3 axillary lymph nodes; N2, Metastases in 4 to 9 axillary lymph nodes. Mutations and copy number alterations (CNAs) of *BCL2L13*, the impact of these alterations on patients’ overall survival (OS), and *BCL2L13* co-expressed genes in kidney cancer were achieved by cBioPortal for cancer genomics studies (http://www.cbioportal.org/) [26]. |Spearman’s r|> 0.4 was set to sort out the *BCL2L13* co-expressed genes in ccRCC and pRCC.

### TargetScan and the encyclopedia of RNA interactomes (ENCORI)

TargetScan (http://www.targetscan.org) was engaged to analyze the potential target microRNA (miRNA) in homo sapiens, conformed to the canonical transcript of *BCL2L13*, which supported by 736 3P-seq tags [27]. Correlation between *BCL2L13* and the target miRNA in kidney cancer were analyzed on ENCORI pan-cancer analysis platform (http://starbase.sysu.edu.cn/panCancer.php), upon the expression data from TCGA [28].

### Cytoscape and Search Tool for the Retrieval of Interacting Genes (STRING)

Kyoto Encyclopedia of Genes and Genomes (KEGG) pathway enrichment analysis and hubs of *BCL2L13* co-expressed genes were accomplished within Cytoscape, with the plug-in ClueGO, CluePedia, MCODE and CytoHubba, which realized by the grid topology algorithm density of maximum neighborhood component (DMNC) [29–31]. STRING database (https://string-db.org/) was combined for sifting the hub genes [32].

### Transient transfection and apoptosis induction

Full-length of human *BCL2L13* was cloned into eukaryotic S-tag fusion expression vector pcDNA3-S-tag via *Eco*RI/*Xho*I (TaKaRa) restriction sites. The recombinant plasmids were then harvested from *Escherichia coli* DH-5α. Transient transfection was performed with Lipofectamine 2000 (Invitrogen, 11668019) according to the manufacturer’s instructions.

HEK293T cells, CAKi-1 cells and 786-0 cells were maintained and grown under a humidified atmosphere (37 ℃, 5% CO_2_), in Dulbecco’s modified Eagle’s medium (Hyclone, SH30243.1), supplemented with 10% fetal bovine serum (HyClone, SH30084.03) and 1% penicillin–streptomycin (Gibco, 15070063). Apoptosis was induced in the transfected 786–0 cells by co-treatment of ABT-263 (navitoclax) (YEASEN, 50804ES08) for 24 h right after transfection.

### Western blotting

Whole cell extract was prepared, followed by western blotting analysis with the following primary antibodies: anti-PARP (Cell Signaling Technology, 9542), anti-S-Tag (Cell Signaling Technology, 12774), anti-BCL2L13 (Santa Cruz Biotechnology, sc-390598), anti-GAPDH (Affinity, AF7021) and anti-β-tubulin (Affinity, AF7011), all with a 1:1000 dilution. Gel image system (Tanon, version 4.2) was used for optical densitometric analysis.

### Immunohistochemistry (IHC)

Paraffin-embedded pathology specimens from 7 patients with ccRCC were used, including renal cell carcinoma tissues and corresponding paracancerous tissues. IHC was carried out following the standard procedure with diaminobenzidine (DAB) detection kit (MXB Biotechnologies, DAB-0031). Anti-BCL2L13 (Proteintech, 16612-1-AP) was diluted 1:100 in 0.1% goat serum PBS solution, incubated at 4 ℃ overnight.

### Apoptotic assay

The optical microscope (Olympus, CKX53) and microcamera (TUCSEN, DigiRetina 16, TCapture version 5.1) were used to capture the morphological changes of 786-0 cells after treatments. For quantitative assessment, 786-0 cells were harvested after treatments and stained with both FITC Annexin V and PI (BD Pharmingen, 556547), then subjected to flow cytometry (Beckman Coulter, CytoFLEX). Annexin V + /PI − , Annexin V + /PI + and Annexin V − /PI + cells were used to represent the types of early apoptotic, late apoptotic and dead cells, respectively.

### Statistical analysis

IBM SPSS statistical software (version 24.0) was used for statistical analysis of the experimental data. All other statistical methods come with the web tools by default. Welch's test was conducted for differential expression of functional mRNA in UCSC Xena. Poisson test was adopted to measure the correlation of a given gene in Open Targets Platform. Transcripts per million (TPM) values and Student's *t* tests were employed to calculate the significance of gene expression divergence between categories in UALCAN. The linear dependence of targeted gene pair was evaluated through Spearman’s correlation coefficient and Pearson's correlation coefficient in cBioPortal. Log-rank test was implemented in both UALCAN and cBioPortal for comparison of survival curves, which displayed as Kaplan–Meier plot. P < 0.05 is considered significant in this entry (*, P < 0.05; **, P < 0.01; ***, P < 0.001).

## Results

### *BCL2L13* mRNA expression is significantly reduced in ccRCC and pRCC

**Fig. 1:**
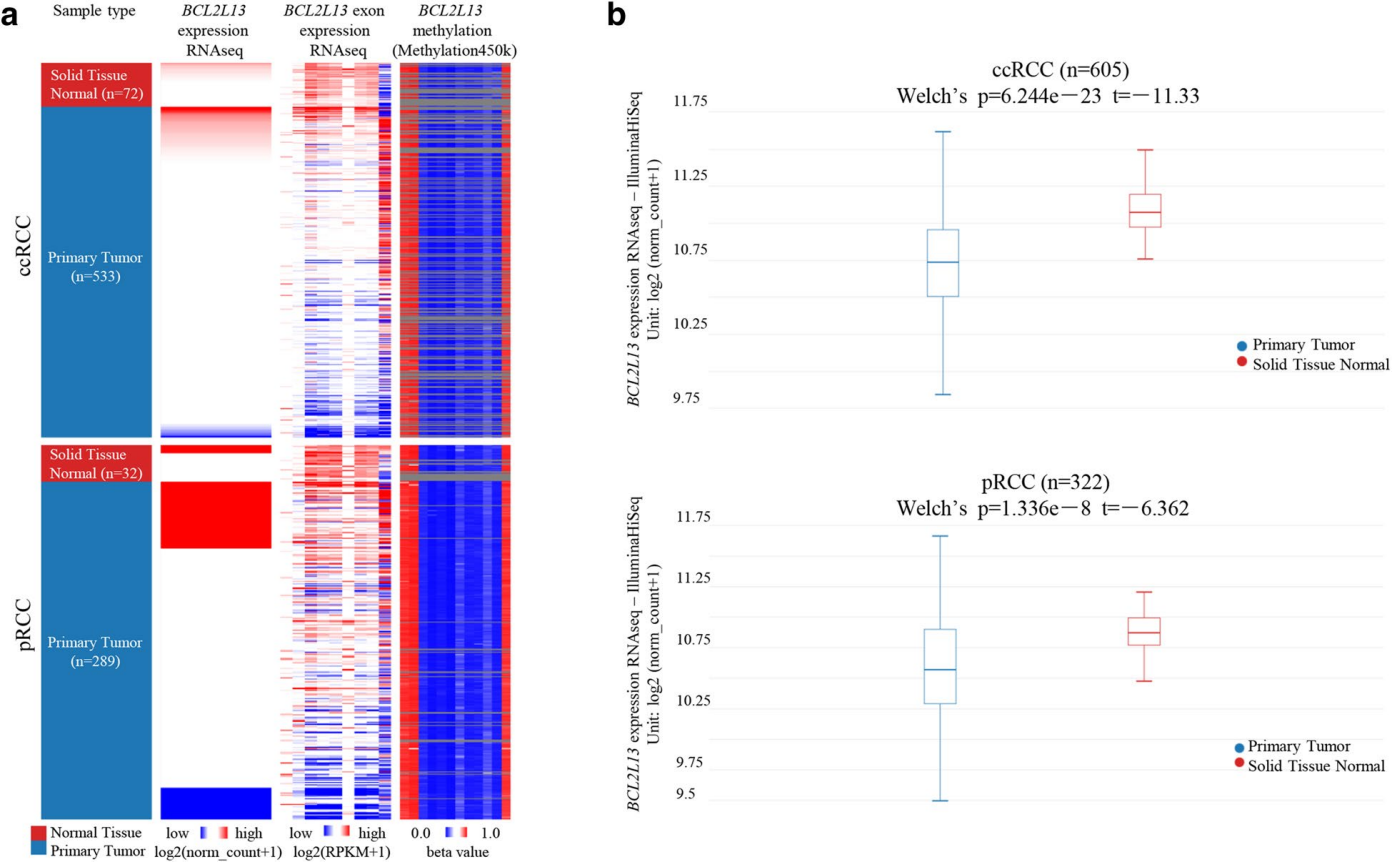
*BCL2L13* mRNA expression is significantly reduced in ccRCC and pRCC, compared to corresponding normal tissue with TCGA data. **A** The heat maps of *BCL2L13* mRNA expression, exon expression and methylation in primary kidney cancer and paracancerous tissue. **B** Down-regulation of *BCL2L13* mRNA is statistically significant in ccRCC (upper) and pRCC (bottom). Welch's *t* test was conducted for the significance

**Fig. 2:**
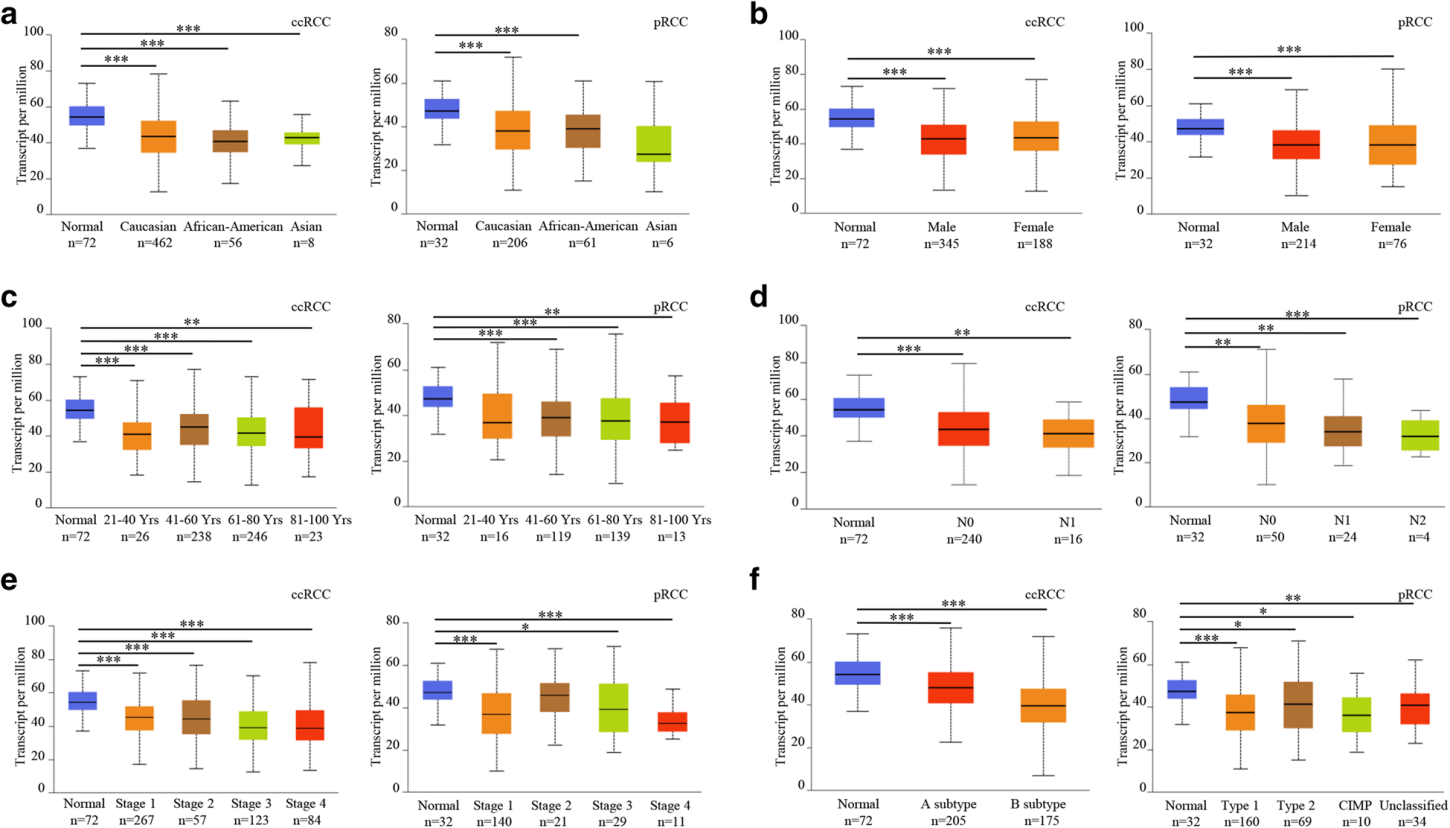
Down-regulated mRNA of *BCL2L13* in ccRCC and pRCC is independent of patients’ race (**A**), gender (**B**), age (**C**), lymph node metastasis status (**D**), clinical stages (**E**) and tumor subtypes (**F**) (CIMP, CpG island methylator phenotype). TPM values and Student's *t* tests were employed to compute the significance of gene expression divergence

*BCL2L13* expresses anomalously in a variety of tumors, participating in tumor progression with its apoptosis-regulating activity [33–35]. The mRNA expression of *BCL2L13* was analyzed in a variety of tumors using the data from TCGA. Significant differences were found between quite a few cancer tissues and their normal counterparts, including BLCA, BRCA, CHOL, COAD, HNSC, chRCC, ccRCC, pRCC, LIHC, LUAD, LUSC, READ, THCA, STAD, UCEC (Additional file 1, Additional file 11). *BCL2L13* mRNA is highly expressed in chRCC, but has no effect on prognosis. In contrast, significant reduction of *BCL2L13* mRNA and exon expression were both found in ccRCC and pRCC primary tumors, when compared to corresponding normal tissues (Fig. 1). Further analysis in ccRCC and pRCC indicated that the reduced *BCL2L13* mRNA is independent of patients’ race, gender, age, lymph node metastasis status, clinical stages and tumor subtypes (Fig. 2, Additional file 1). Moreover, down-regulation of *BCL2L13* actively impact on the patients’ survival probability (see below). In other cancers not mentioned herein, the expression differences between tumor tissues and normal tissues were not significant, which might succumb to the corresponding normal tissues are scarcity in TCGA database.

The clinical proteomic tumor analysis consortium (CPTAC) was then engaged to analyze the protein expression level of *BCL2L13* in renal cell carcinoma. The *BCL2L13* protein expression is consistently lower in ccRCC patients compared to healthy crowd, which is independent of the tumor stage (Additional file 2). However, the relevant data is unavailable for pRCC.

Abnormal methylation of genes often plays an important role in cancer progression. DNA methylation of *BCL2L13* coding regions was not altered in renal cell carcinoma samples (Fig. 1A). Moreover, *BCL2L13* promoter methylation was elevated significantly in ccRCC, but not in pRCC (Additional file 3). Specifically, hypermethylation of *BCL2L13* promoter in ccRCC is independent of patients’ age, gender, race, clinical stages, lymph node metastasis status and tumor grade (Additional file 3).

### Low expression of *BCL2L13* has significant impact on survival probability in ccRCC and pRCC

**Fig. 3:**
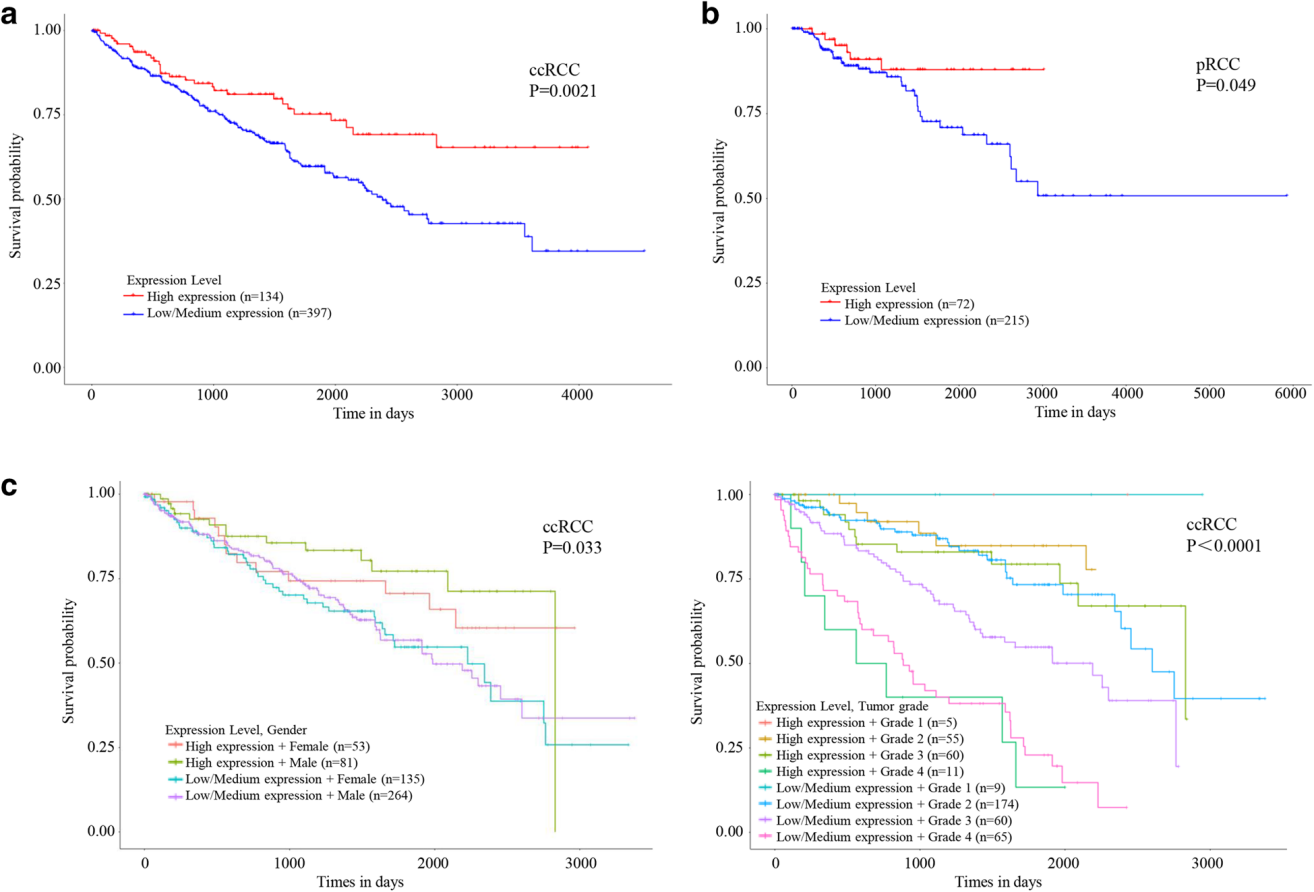
Low expression of *BCL2L13* significantly affects the prognosis of ccRCC and pRCC. **A**–**B** Kaplan–Meier survival plot of ccRCC (**A**) and pRCC (**B**) that grouped by *BCL2L13* mRNA levels. **C** Poor prognosis of ccRCC that related to the down-regulation of *BCL2L13* is independent of patients’ gender (left) or tumor grade (right). Log-rank test was implemented for comparison of survival probability

To evaluate how *BCL2L13* expression impact on patient survival, ccRCC and pRCC cases were grouped by their *BCL2L13* mRNA levels. *BCL2L13* low expression significantly correlated with poor prognosis in ccRCC (P = 0.0021, Fig. 3A), which was independent of patients’ gender or tumor grade (Fig. 3C). In pRCC, similar results were observed, albeit to a lesser extent (P = 0.049, Fig. 3B). *BCL2L13* expression levels had no obvious impact on patient survival of other cancer types aforementioned by UALCAN (Additional file 4).

Somatic mutations and gene CNAs could also influence the prognosis of cancer patients [36]. Through analysis using data from TCGA, *BCL2L13* copy number altered only 0.2% in ccRCC with deep deletion, and 1.1% in pRCC with deep deletion and missense mutations (Additional file 5). Amino acid mutations of BCL2L13 were only found in pRCC cases, namely, Ser38 was mutated to Leu and Gly182 was mutated to Ser, in which S38L occurred mainly in the patients with shallow deletion of *BCL2L13* copy number (Additional file 5). *BCL2L13* maintained diploid status in ccRCC and pRCC, while the other genetic changes (gain, shallow/deep deletion) were found (Additional file 5). Because only a few patients carry *BCL2L13* genetic changes, the impact of these changes on prognosis of ccRCC and pRCC is undeterminable due to limited patient numbers (Additional file 6).

### *BCL2L13* doesn’t correlate with the kidney cancer related genes or putative target miRNA in ccRCC and pRCC

**Fig. 4:**
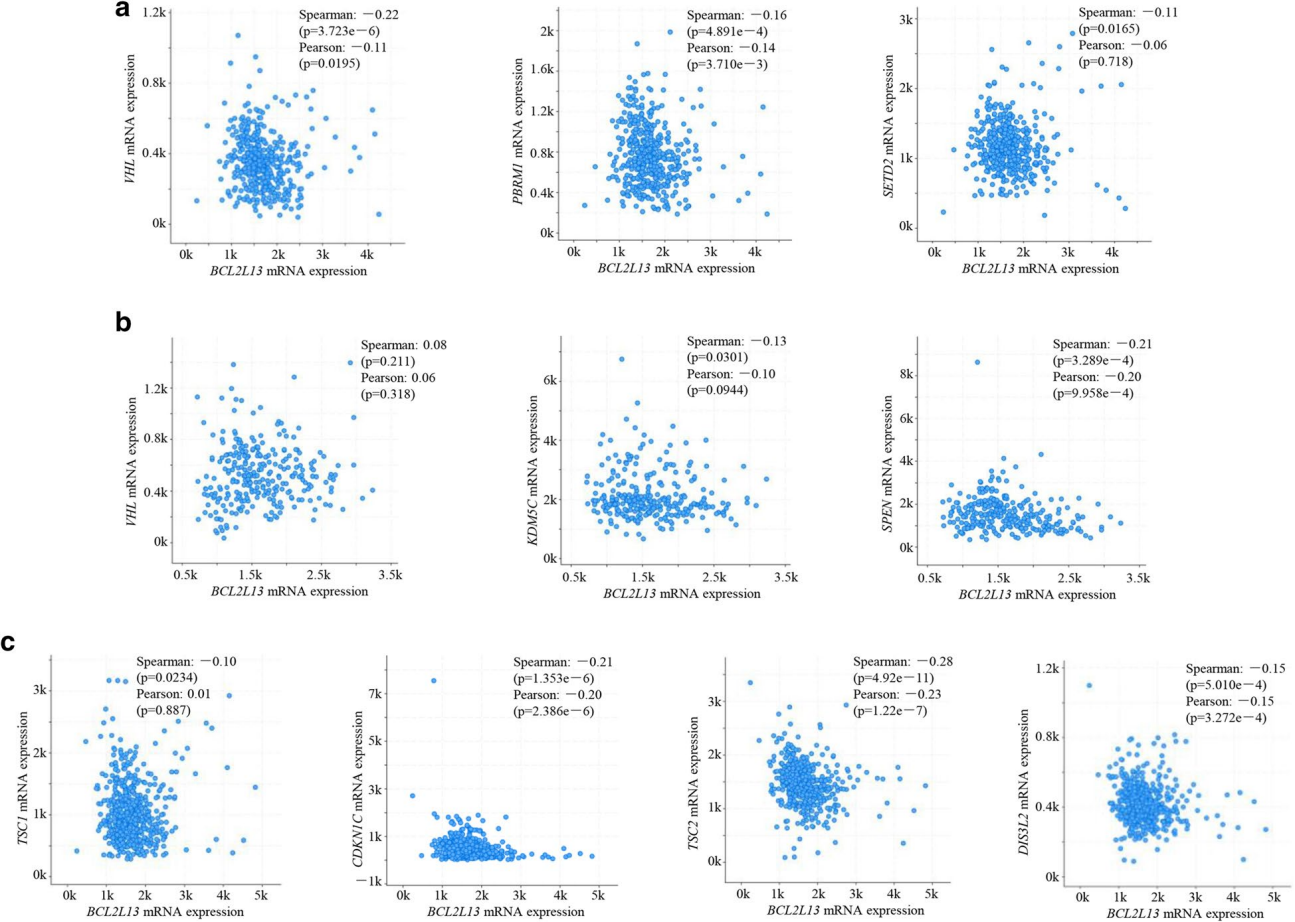
*BCL2L13* showed low correlation with the kidney cancer related genes. **A**–**B** Correlation between *BCL2L13* and the top 3 mutated genes in ccRCC (**A**) or pRCC (**B**). **C** *BCL2L13* had low correlation with the top 5 genes associated with inherited kidney cancer-predisposing syndrome (for *VHL* shown in **A**). The linear dependence was evaluated by both Spearman’s correlation and Pearson's correlation analysis

Previous studies have found that several genes, including *VHL*, *PBRM1* and *TSC1*, played an important role in tumorigenesis of kidney cancer [37]. To uncover the mechanisms underlying the aggravated poor prognosis drived by *BCL2L13* downregulation in ccRCC and pRCC, the correlation between *BCL2L13* and these genes were analyzed. *BCL2L13* correlated weakly with the most frequently mutated *VHL*, *PBRM1*, *SETD2* in ccRCC and *VHL*, *KDM5C*, *SPEN* in pRCC respectively (Fig. 4, Additional file 7). Hereditary kidney cancer patients occupy 5–8% in all the diagnosed ones, and commonly harbor some cancer predisposition genes, such as *TSC1/2*, *CDKN1C* and *DIS3L2* [38, 39]. *BCL2L13* showed frail correlation with the top 5 genes *TSC1/2*, *CDKN1C*, *VHL* and *DIS3L2*, that associated with inherited kidney cancer-predisposing syndrome (Additional file 12, Fig. 4C) [40].

*BCL2L13* can be regulated by miRNA, such as miRNA-874-3p, miRNA-124 and -137 [41–43]. Therefore, the correlation between miRNA and *BCL2L13* in kidney cancer were analyzed. Those reported candidate miRNA, however, only have weak correlation with *BCL2L13* (Additional file 13).

While only weak correlation was uncovered between *BCL2L13* and the known kidney cancer related genes, we found that voltage-dependent L-type calcium channel subunit beta-1 (*CACNB1*) and numb-like protein (*NUMBL*) are the two *BCL2L13* negatively correlated genes. Compared to the downregulation of *BCL2L13*, *CACNB1* and *NUMBL* were both significantly upregulated in ccRCC and pRCC, and also affected the prognosis of these patients (Additional file 8).

### *BCL2L13* regulates metabolism pathway in ccRCC and pRCC

BCL2L13 has previously reported to participate in several physiological processes. For example, down-regulated BCL2L13 inclined to relieve brain injury induced by ischemia/reperfusion (I/R) [42]. Mitochondrial dynamics and biogenesis, which is also regulated by BCL2L13, could facilitate the browning process of preadipocytes [44]. Moreover, low expression of BCL2L13, which was related to weakened oxidative phosphorylation and enhanced glycolysis, was often found in cancer cells [45, 46]. Thus, the cellular functions of *BCL2L13* might have latent impact on the poor prognosis of ccRCC and pRCC.

**Fig. 5:**
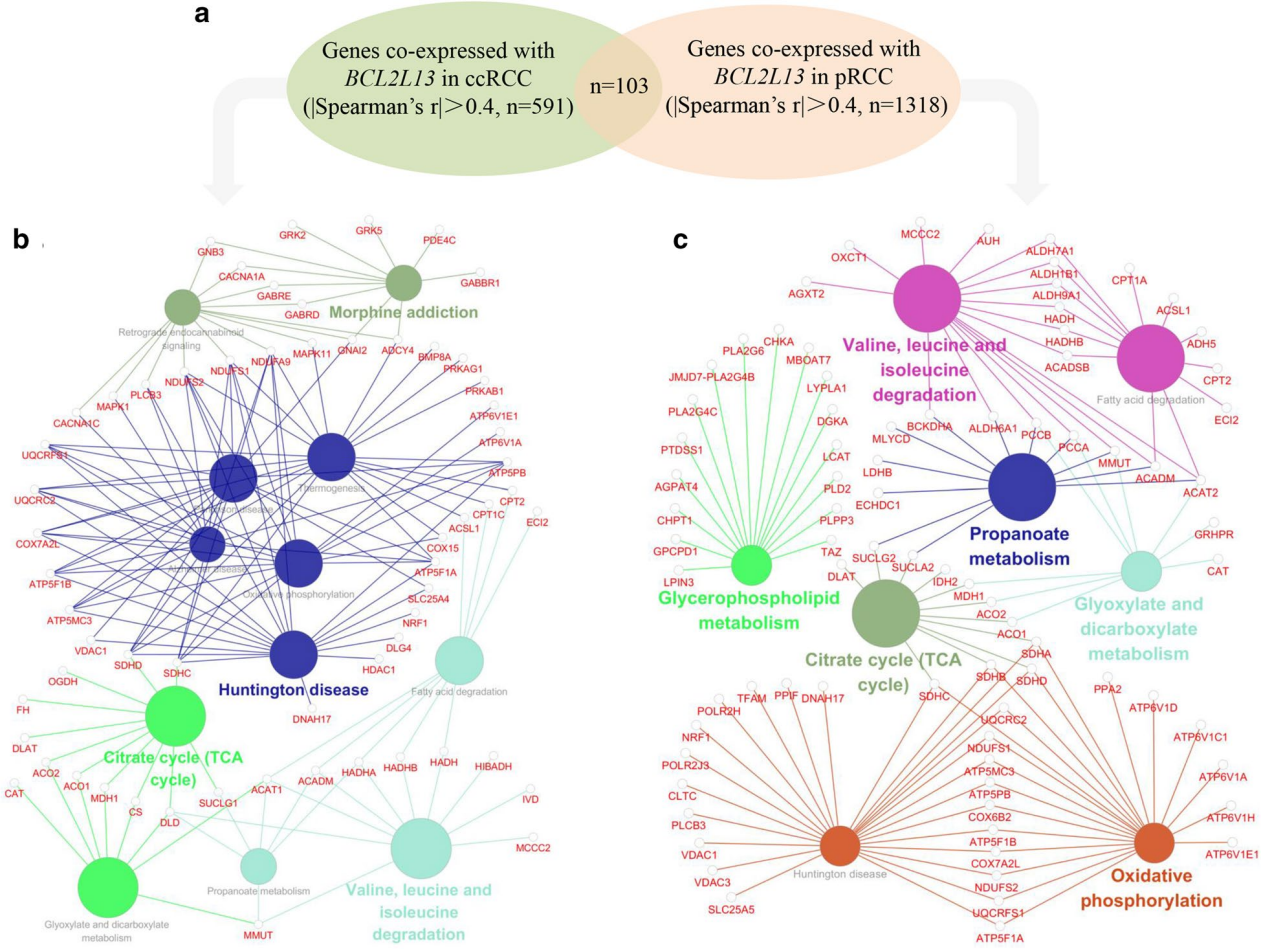
Enrichment analysis of *BCL2L13* co-expressed genes in ccRCC and pRCC. **A** Co-expressed genes of *BCL2L13* were sorted out by |Spearman’s r|> 0.4, 591 target genes in ccRCC and 1318 target genes in pRCC were found respectively. **B**–**C** KEGG pathway enrichment analysis of *BCL2L13* co-expressed genes in ccRCC (**B**) and pRCC (**C**), filtered by P ≤ 0.05

Co-expressed genes of *BCL2L13* were investigated for comprehensive grasp [47]. Filtered by |Spearman’s r|> 0.4, 519 and 1318 *BCL2L13* co-expressed genes were found in ccRCC and pRCC respectively (Fig. 5A, Additional file 14). KEGG pathway enrichment analysis manifested the physiological characteristics of these genes, including Huntington disease, citrate cycle and substance metabolism for ccRCC (Fig. 5B), and oxidative phosphorylation, citrate cycle, substance metabolism and Huntington disease for pRCC (Fig. 5C). No substantial differences were found between ccRCC and pRCC. While many of these pathways of *BCL2L13* co-expressed genes are related to citrate cycle and substance metabolism, these data suggested that *BCL2L13* regulated energy and metabolism might be involved in its prognostic role in ccRCC and pRCC patients. ccRCC was selected for further mechanism exploration, because of the more significant prognostic role of *BCL2L13* low expression in this kind of cancer.

### *BCL2L13* has positive correlation with *SLC25A4* (ANT) in ccRCC

**Fig. 6:**
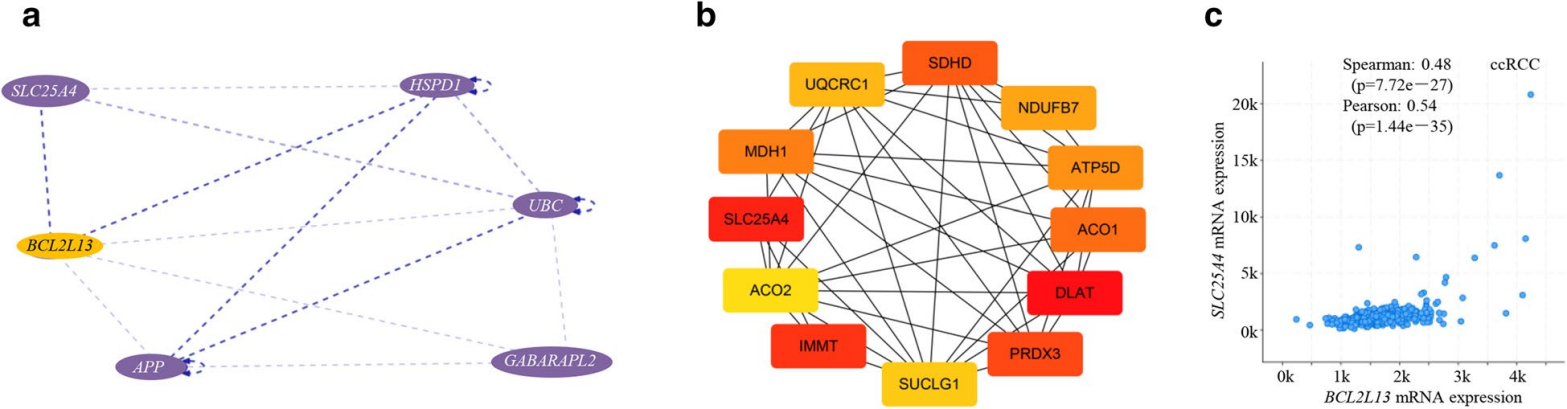
*BCL2L13* exhibits strong correlation with *SLC25A4* (ANT). **A** Protein–protein interaction network analysis of BCL2L13. **B** Hubs of *BCL2L13* co-expressed genes in ccRCC. **C** *BCL2L13* has high correlation with *SLC25A4* in ccRCC. The linear dependence was evaluated by Spearman’s correlation and Pearson's correlation analysis

The PPI network and hub genes were analyzed for BCL2L13 regulated metabolism, and a small-scale of PPI network was found based on this analysis (Fig. 6A). First, a PPI of BCL2L13 and HSP60 (heat shock protein 60, encoded by *HSPD1*) was found, because BCL2L13 is anchored on the outer mitochondria membrane, while HSP60 is distributed in the mitochondrial matrix, resulting in a fluorescence co-localization [17]. Second, BCL2L13 had PPI connections with UBC, GABARAPL2 and APP respectively, implying that it may engaged in mitophagy [48–51]. Third, BCL2L13 was reported to interact with ANT, the protein localized in mitochondrial inner membrane. DMNC, which can be employed for some covert hubs, was further used for the essential genes calculating in Cytoscape, for its higher hit rate to key nodes compared to general degree method [52]. Through this analysis, *SLC25A4* was found to be one of the senior hub genes that mediate physiological activity of *BCL2L13* (Fig. 6B).

ANT is in charge of exchanging ADP for ATP through MPTP, executing a fundamental role in mitochondrial respiration [53]. ANT could induce mitophagy independent of its ADP/ATP exchange activity, though inhibition or ablation of ANT culminate in disparate phenotypic mitophagy [54]. Studies have evinced that ANT substantially interacts with BCL2L13, mediating its pro-apoptotic activity, while further analysis also supported their correlation (Fig. 6C) [17, 55, 56].

### BCL2L13 overexpression promote apoptosis in ccRCC cells 786-0

**Fig. 7:**
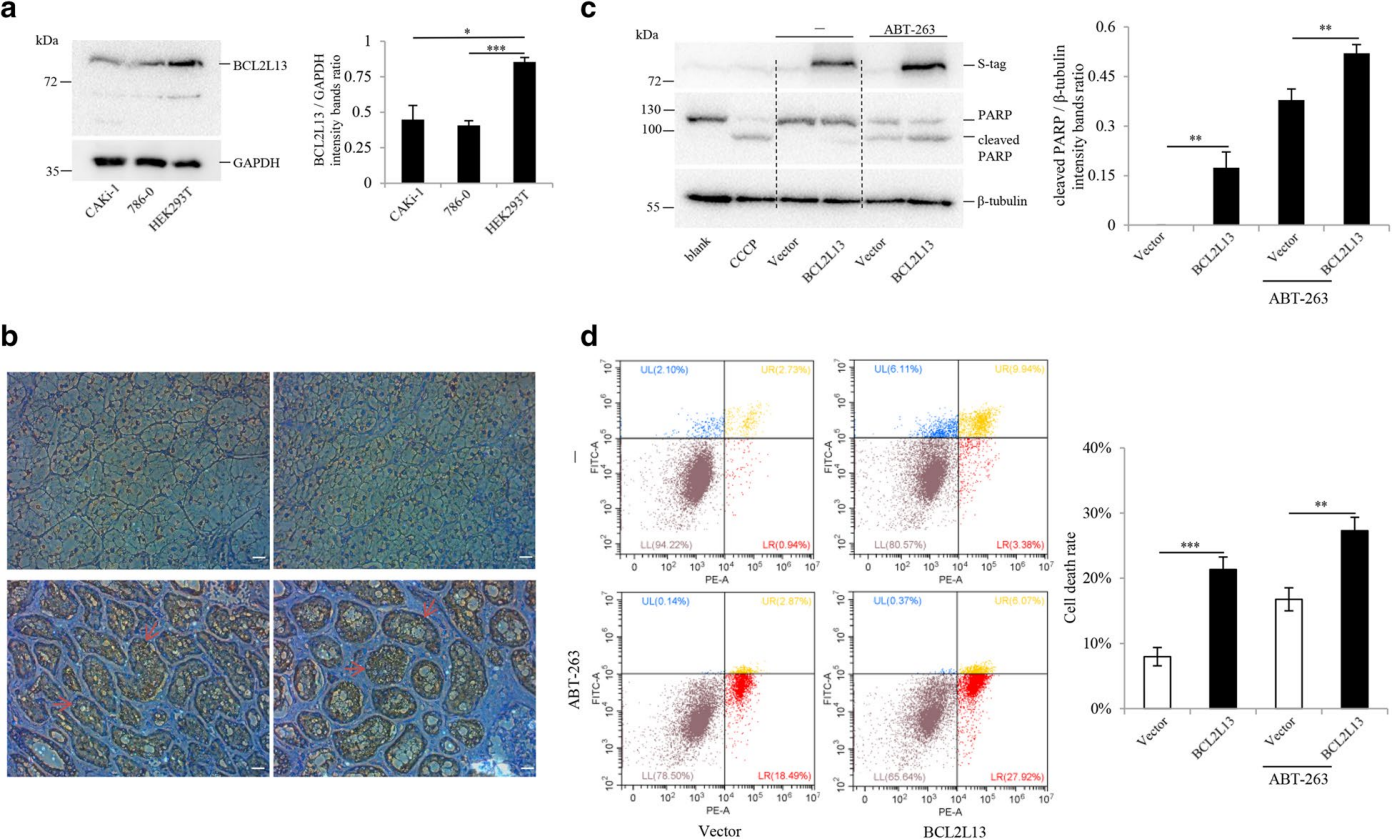
*BCL2L13* performs pro-apoptotic function in ccRCC cell line 786-0. **A** *BCL2L13* expression is conspicuously lower in CAKi-1 cells and 786-0 cells, compared to HEK293T cells. **B** *BCL2L13* expression is significantly lower in ccRCC tissues (upper row), compared to paracancerous tissues (lower row), when glomeruli (red arrows) are stained. Scale bar: 20 μm. **C**–**D** *BCL2L13* overexpression promotes apoptosis in 786-0 cells. 786-0 cells were transfected with indicated genes and treated with ABT-263 (5.5 μM) for 24 h, then subjected to western blotting (**C**) or flow cytometry (**D**). 786-0 cells treated with CCCP (50 μM) for 24 h were shown as a positive control (2nd lane of panel **C**). Results are shown as the mean ± s.e.m. **A** one-way analysis of variance (ANOVA) was performed by SPSS. * P < 0.05

786-0 cells were used to study the pro-apoptotic activity of BCL2L13. Compared to HEK (human embryonic kidney) 293 T cells, the expression of BCL2L13 is much lower in clear cell renal cell carcinoma cell line 786-0 (Fig. 7A), consistent with the silico-based analyses. Moreover, the IHC results also indicated a low expression of BCL2L13 in tumor tissues (Fig. 7B). After transient transfection for 48 h, BCL2L13 induces apoptosis in 786-0 cells, characterized by increased cleavage of poly-(ADP-ribose) polymerase (PARP) and also the percentage of Annexin V/PI positive cells (Fig. 7C–D, Additional files 9, 10). BCL2/BCLx_L_/BCLw inhibitor ABT-263 was used to induce mitochondrial mediated apoptosis [57]. Under the treatment of ABT-263 on *BCL2L13* overexpressed 786-0 cells, cleaved PARP and the proportion of Annexin-V/PI positive cells were more apparent (Fig. 7C–D, Additional files 9, 10). These data hint that BCL2L13 performs pro-apoptotic function in ccRCC cells.

## Discussions and conclusions

BCL2L13 belongs to BCL2 protein family, and possesses complete BCL2 homology (BH) 1–4 domains. The BHNo domain embedded between BH regions and the C-terminal transmembrane motif endows BCL2L13 with some non-canonical characteristics [58–60]. BCL2L13 was reported to induce mitochondria fragmentation that in favor of the caspase activation cascade [55]. In addition, BCL2L13 has been shown to accelerate mitophagy by binding to microtubule-associated protein 1 light chain 3 (MAP1LC3), suggesting a role as a mitophagy receptor for the quality control of mitochondria [60, 61]. BCL2L13 is reported to promote apoptosis, but this function doesn’t relate to any of its four BH domains [14, 17]. The pathological action of BCL2L13 will be the next focal point in this specialism.

Declining *BCL2L13*-directed poor prognosis in ccRCC is independent of patients’ gender or tumor grade. On the other hand, in ccRCC and pRCC, *BCL2L13* has weak correlation with the genes mutated in kidney cancer or the genes associated with inherited kidney cancer predisposing syndrome [62, 63], as well as the *BCL2L13* related miRNA. That miRNA may not the proximate cause of reduced *BCL2L13* in cancerous renal tissues. Although *BCL2L13* does not show strong correlation with these genes or miRNA, *SLC25A4* (ANT) exhibits high correlation with *BCL2L13*, supposed to play as one of the hubs involved in *BCL2L13*-mediated prognostic consequence of kidney cancer [17]. And the attenuated BCL2L13-ANT pathway may be a possible reason for poor prognosis of ccRCC, that is different from its performance in GBM cells [15].

In addition, less genetic alterations of *BCL2L13* in ccRCC and pRCC are observed, complied with the data from TCGA, suggesting that *BCL2L13* is relatively genetic stable in kidney cancer patients. Shallow deletion of the copy number in a fraction of patients may result in the low expression of *BCL2L13* in pRCC, while promoter hypermethylation of *BCL2L13* probably accounts for the low expression in ccRCC. Similar functions of *BCL2L13* are presented in ccRCC and pRCC by KEGG pathway enrichment analysis. However, it remains an attractive hypothesis that *BCL2L13* maintains a linear dose–effect relationship with its physiological activity.

Taken together, *BCL2L13* may act as an independent and desirable prognostic marker for ccRCC and pRCC. In addition to that, the functions of *CACNB1* and *NUMBL* seem antagonistic toward *BCL2L13* activity, while further experimental trial on them may provide new remedies for renal cancer patients.

## Supplementary Information

**Additional file 1:** *BCL2L13* mRNA expression was evaluated in pan-cancer and corresponding normal tissues, including chRCC, ccRCC and pRCC (A). *BCL2L13* mRNA expression is significantly reduced, independent of tumor grade in ccRCC (B) or body mass in pRCC (C). Student's *t* tests were employed to calculate the P value.

**Additional file 2:** Protein expression of BCL2L13 was evaluated in several tumor types (A). It is significantly reduced in ccRCC (B), independent of tumor stage (C), with the data from CPTAC. Student's *t* tests were employed to calculate the P value.

**Additional file 3:** *BCL2L13* promoter methylation is significantly elevated in ccRCC, but not in pRCC (A). Hypermethylation of *BCL2L13* promoter in ccRCC is independent of patients’ age (B), gender (C), race (D), clinical stages (E), lymph node metastasis status (F) and tumor grade (G). Student's *t* tests were employed to calculate the P value.

**Additional file 4:** *BCL2L13* has no effect on survival of BLCA, BRCA, CHOL, COAD, HNSC, chRCC, LIHC, LUAD, LUSC, READ, THCA, STAD and UCEC, evaluated by UALCAN. Log-rank test was implemented for the significance.

**Additional file 5:** Genetic alterations of *BCL2L13* occurred in ccRCC (A, upper) and pRCC (A, bottom). 2 missense (variants of uncertain significance, VUS) somatic mutations of BCL2L13 occurred in a few pRCC patients (B). Copy-number alterations of *BCL2L13* in ccRCC (upper) or pRCC (bottom) were supported by GISTIC (genomic identification of significant targets in cancer), mutations were shown as indicated (C).

**Additional file 6:** Kaplan–Meier survival plot of ccRCC (left) and pRCC (right) that grouped by *BCL2L13* mutations & copy number alterations.

**Additional file 7:** Top 20 most frequently mutated genes in ccRCC (left) and pRCC (right).

**Additional file 8:** *CACNB1* and *NUMBL* were both significantly upregulated in ccRCC (A) and pRCC (B), and affect the prognosis of patients (C, D), analyzed by UALCAN with the data from TCGA. Student's *t* tests were employed to compute the P value.

**Additional file 9:** Status of 786–0 cells were captured under microscope (× 10 objective): transient transfection of vehicle for 48 h (A), transient transfection of *BCL2L13* for 48 h (B), vehicle transient transfection followed by ABT-263 (5.5 uM) treatment for 24 h (C) and *BCL2L13* transient transfection followed by ABT-263 (5.5 uM) treatment for 24 h (D).

**Additional file 10:** Uncropped gel images from Fig. 7 A and 7C.

**Additional file 11:** Expression of *BCL2L13* in pan-cancer.

**Additional file 12:** Top 25 genes associated with inherited kidney cancer predisposing syndrome.

**Additional file 13:** Correlation of the target miRNA and *BCL2L13* in ccRCC, pRCC.

**Additional file 14:** *BCL2L13* co-expressed genes in ccRCC, pRCC.

## Data Availability

The data that support the findings of this study were obtained from open access database indicated in “Materials and Methods”, all the data are available from the corresponding author upon reasonable request.
